# A Narrative Review of Independent Treatment Methods for ED: Assessment of the Effectiveness of Diet, Supplements, Pharmacotherapy, and Physiotherapy

**DOI:** 10.3390/jcm14072386

**Published:** 2025-03-31

**Authors:** Marta Bonarska, Damian Adasik, Simone Szymczyk, Gabriela Łocik, Elżbieta Bumbul-Mazurek, Piotr Marianowski, Artur Ludwin

**Affiliations:** 1 1st Department of Obstretrics and Gynaecology, University Centre for Women and Newborn Health, pl. Starynkiewicza 1/3, 02-015 Warsaw, Poland; bonarskam99@gmail.com (M.B.); aadasik97@gmail.com (D.A.); gabriela.locik@gmail.com (G.Ł.); ela.bumbul@gmail.com (E.B.-M.); pmarianowski@wum.edu.pl (P.M.); artur.ludwin@wum.edu.pl (A.L.); 2 Faculty of Medicine, Medical University of Warsaw, 61 Zwirki i Wigury Street, 02-091 Warsaw, Poland

**Keywords:** erectile dysfunction, shock wave therapy, diet, regenerative medicine, pelvic floor muscle exercises, manual therapies, Mediterranean diet, ginseng, yohimbine, epimedium, penile prostheses

## Abstract

The prevalence of erectile dysfunction (ED) among the male population worldwide has significant ramifications for their quality of life and psychological well-being. This narrative review explores both conventional treatments, such as pharmacotherapy and surgery, and emerging approaches, including regenerative therapies, dietary interventions, physiotherapy, and vacuum erection devices (VEDs). Unlike prior reviews, this study emphasises unconventional therapies and their role in comprehensive ED management. A systematic literature review was conducted using PubMed, Embase, and Medline, including studies published up to 2024. Keywords such as “ED”, “pharmacotherapy”, “shock wave therapy”, “regenerative medicine”, and “dietary interventions” were used to identify relevant studies. Eligible studies examined treatment efficacy, mechanisms, and patient outcomes. Phosphodiesterase type 5 (PDE5i) inhibitors remain the primary treatment, demonstrating effectiveness across diverse populations. Regenerative therapies, including stem cells and platelet-rich plasma (PRP), show promise, but require further validation. Surgical interventions, particularly penile prostheses, provide high patient and partner satisfaction. Non-invasive methods, including physiotherapy and dietary changes like adoption of the Mediterranean diet, improve vascular health and erectile function. The efficacy of VEDs as standalone or adjunct treatments has been demonstrated, enhancing outcomes in prosthetic surgery. A multimodal, personalised approach is essential for optimising ED treatment. Despite promising advancements, gaps remain in terms of long-term data, standardised protocols, and partner-centred outcomes. Future research should focus on large-scale, multi-centre trials and synergistic treatment approaches to improve therapeutic outcomes and patient satisfaction.

## 1. Introduction

Erectile dysfunction (ED) is defined as a persistent inability to achieve or maintain sufficient penile rigidity for satisfactory sexual performance [1]. This condition affects millions of men worldwide, significantly impacting their quality of life and contributing to psychological distress and diminished interpersonal relationships. This can result in decreased self-esteem, anxiety, and strained relationships [2,3,4]. The overall prevalence of ED (ED) was 22%, with rates increasing with age across all racial and ethnic groups [5].

It is reported that 22.7% of men who seek assistance for ED are aged 40 years or younger [6]. A mere 23% of men received efficacious ED treatments [7]. This complex disorder has a number of potential causes, including vascular, neurological, hormonal, and psychological disturbances [4]. Psychosocial factors were more prevalent among younger men (in their 20s and 30s), while organic factors, such as hypertension and diabetes, were more influential among older men [8]. Among diabetic men, the prevalence of ED was 58.5%, with 40.9% classified as moderate and 6.8% as severe cases [9]. Strong correlations were observed between age, duration of diabetes, and type of medication [9].

ED is most commonly understood as a vascular disorder, in which impaired blood flow to the penile tissue plays a pivotal role. Research has demonstrated that conditions such as atherosclerosis, cardiovascular diseases, hypertension, and diabetes significantly contribute to the prevalence of ED.

Furthermore, neurological conditions that affect the nerve signals required for achieving and maintaining an erection also play a crucial role in the development of ED [10]. The treatment landscape for ED has historically been dominated by pharmacological therapies, particularly phosphodiesterase type 5 inhibitors (PDE5i), such as sildenafil and tadalafil [1,4]. However, due to contraindications and the occurrence of adverse effects, such as headaches, visual disturbances, flushing, hypotension, and dizziness, these medications cannot be used by all patients [4,11,12]. This situation has resulted in research into alternative therapeutic options, including lifestyle interventions, psychological counselling, physical therapy, hormonal therapy, vacuum erection devices, surgical methods including penile prosthesis, and innovative regenerative methods, such as stem cell therapies and platelet-rich plasma [13]. Each of these treatments offers distinctive benefits and can be adapted to suit the specific requirements of individual patients, emphasising the value of a comprehensive and multidisciplinary approach to ED management [14]. Recent research has highlighted the influence of lifestyle and diet on ED. Dietary patterns have been associated with a reduced risk of ED due to their cardiovascular benefits. Nutrients such as nitrate-rich vegetables, fruits, whole grains, and healthy fats have been identified as playing a pivotal role in maintaining endothelial health and influencing hormonal balance, which are essential for optimal erectile function [15].

Additionally, regular physical activity and moderate alcohol consumption have been demonstrated to contribute to a reduction in the severity of ED. The objective of this review is to examine the range of treatment options for ED, with a particular focus on their efficacy, mechanisms of action, and potential integration into a comprehensive treatment plan. The aforementioned review should facilitate the creation of a personalised treatment plan for patients diagnosed with this disorder.

## 2. Materials and Methods

**Figure 1:**
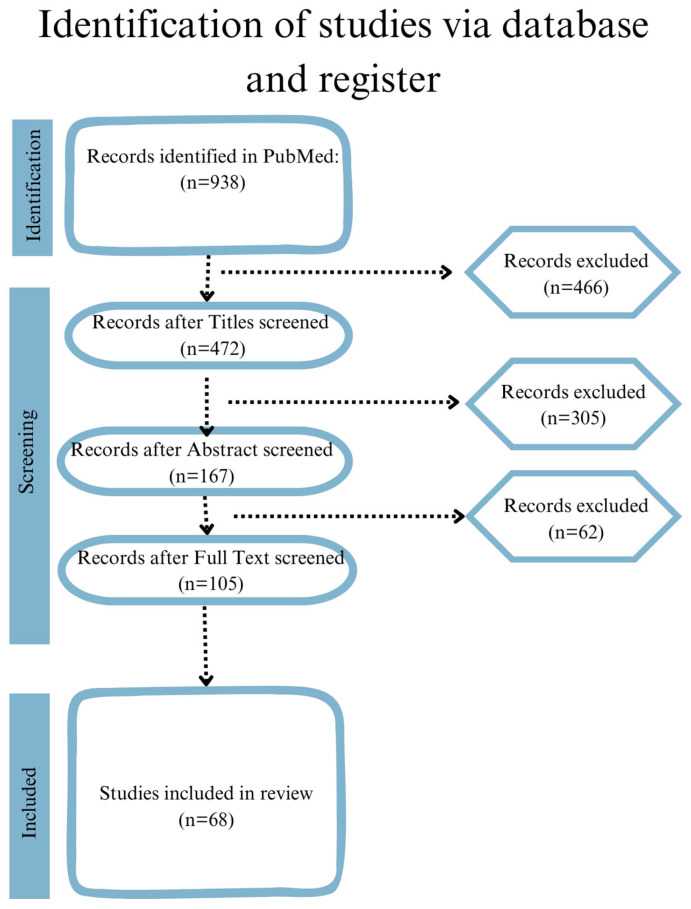
Flow diagram of study selection process.

A comprehensive literature search was conducted between March and November 2024, using the PubMed, Embase, Medline, and ProQuest databases. The objective of the search strategy was to identify studies that explore various methods of treatment for ED. The search terms included a combination of Medical Subject Headings (MESH) and free-text terms, such as “ED”, “surgical methods”, “treatment”, “sexual satisfaction”, “supplements”, “vacuum erection devices”, “diet”, “penile prosthesis”, “pharmacotherapy”, “shock wave therapy”, “gene therapy”, and “physiotherapy”. These terms were used in conjunction with one another term and independently. To ensure the quality of the studies included in this review, two independent reviewers evaluated the results of the preliminary search. The selection procedure entailed an evaluation of the studies’ complete text, abstract, and title to ascertain their suitability and contribution to the review’s objectives. To be considered for inclusion in the review, studies were required to address the treatment of ED and provide information on the therapy’s effects, any associated side effects, and the underlying physiological mechanisms. Book chapters, duplicates, and conference proceedings were excluded from the analysis. In this study, ChatGPT 4.0 and Endnote were employed to provide citations. Furthermore, ChatGPT 4.0 and Grammarly were utilised for grammar and spelling checks, ensuring clarity and precision in our writing. A flow diagram of the study selection process is illustrated in Figure 1. The reviewed studies are presented in Table S1. The methodological rigor of the included studies was systematically assessed using appropriate quality evaluation tools. For randomised controlled trials (RCTs), the Cochrane Risk of Bias (RoB 2) tool was applied to evaluate six domains: the randomisation process, deviations from the intended interventions, missing outcome data, measurement of the outcome, selection of the reported result, and overall bias judgment. Studies were classified as low risk, some concerns, or high risk of bias, based on these criteria. For observational studies, the ROBINS-I (Risk of Bias in Non-Randomized Studies of Interventions) tool was employed to evaluate bias across seven domains: bias due to confounding, selection of participants, classification of interventions, deviations from the intended interventions, missing data, measurement of outcomes, and selection of reported results. Studies were categorised as having low, moderate, serious, or critical risk of bias. Studies with high or critical risk of bias were excluded unless they offered unique insights pertinent to the treatment of emergency department patients. Furthermore, the reliability of the studies was assessed by evaluating the sample size adequacy, statistical power, and generalisability. Notable limitations identified in the extant literature included heterogeneity of the outcome measures and a paucity of standardised reporting, which hindered cross-study comparisons and meta-analyses.

## 3. Discussion

The existing literature on treatments for ED offers valuable insights; however, several methodological shortcomings restrict the robustness and generalisability of the findings. A significant number of studies, particularly those examining regenerative therapies (such as stem cell therapy and PRP), lack the longitudinal follow-up necessary to assess the sustained efficacy and safety of these treatments. It is recommended that future research includes extended follow-up periods (e.g., >5 years) in order to evaluate the long-term outcomes, durability, and potential adverse effects of novel treatments. The reliance on small participant cohorts in regenerative and dietary studies often results in a reduction in statistical power and a limitation in regard to the generalisability of the findings. It is imperative that larger, multi-centre randomised controlled trials (RCTs) are conducted to confirm the findings and address population diversity. The existence of disparities in outcome measures, patient populations, and methodologies, as well as, for example, the lack of placebo control, presents a significant obstacle to conducting meta-analyses and comparisons. The implementation of standardised protocols for ED research, including the consistent use of validated scales, such as the International Index of Erectile Function (IIEF), would facilitate greater comparability. A paucity of studies address the issue of partner satisfaction or the psychosocial dynamics of ED treatment. It is recommended that future research incorporates partner-reported outcomes and relationship assessments in order to provide a comprehensive view of the therapeutic impact. It is common practice in studies to exclude patients with significant comorbidities (e.g., diabetes, cardiovascular disease), despite these being common in ED populations. It would be beneficial for future trials to include patients with comorbid conditions in order to reflect real-world populations and assess differential treatment responses. Regenerative therapies, while promising, rely heavily on preclinical data or early-phase trials with inconsistent methodologies. In order to validate regenerative interventions and establish standard protocols, it would be beneficial for future studies to employ rigorous, double-blind, placebo-controlled trials. Additionally, the management of ED (ED) in patients with gastrointestinal (GI) disorders is often overlooked [16]. A nationwide survey conducted by the Italian Society of Gastroenterology (SIGE) revealed that sexual dysfunction, including ED, is prevalent among patients with GI or liver conditions [16]. However, gastroenterologists frequently do not address these issues due to factors such as a lack of knowledge, time constraints, and embarrassment [16]. The study identified that the predominant sexual dysfunction reported by male patients with GI or liver conditions was ED, constituting 75% of the cases [16]. This underscores the necessity for heightened awareness and education among gastroenterologists to incorporate sexual health into routine care, ensuring comprehensive management of patients with GI disorders. Moreover, recent research has demonstrated a robust association between obstructive sleep apnoea (OSA) and ED, suggesting that underlying hypoxia and vascular dysfunction may contribute to erectile impairment [17]. A study revealed that 68.1% of patients with severe OSA experienced ED, with worsening symptoms associated with lower oxygen saturation levels [17]. This underscores the necessity for a multidisciplinary approach to the treatment of ED, particularly in patients with comorbid conditions such as OSA, metabolic syndrome, and cardiovascular disease. Furthermore, the potential impact of lifestyle factors, such as physical activity, on the management of ED merits further investigation. Regular exercise has been associated with improved erectile function, potentially due to enhanced cardiovascular health and endothelial function. Incorporating lifestyle modifications, alongside medical treatments, could offer a more holistic approach to ED management.

### 3.1. Pharmacotherapy

PDE5i represent the foundation of contemporary pharmacological treatment for ED. These agents have been developed to specifically target the phosphodiesterase 5 enzyme, which is primarily located in the erectile tissue of the penis, as well as in the pulmonary vasculature [12,18]. By inhibiting this enzyme, PDE5i promote the relaxation of smooth muscle cells and increase blood flow to the penis, thereby facilitating the achievement and maintenance of an erection in response to sexual stimulation [4,12]. PDE5i function by selectively inhibiting the breakdown of cyclic guanosine monophosphate (cGMP), a molecule that plays a crucial role in the nitric oxide (NO) signalling pathway [4,12,19]. During the process of sexual arousal, NO is released from nerve endings and endothelial cells in the penis, which stimulates the production of cGMP [4,12,19,20]. Increased levels of cGMP result in the relaxation of smooth muscle tissue within the corpus cavernosum, thereby facilitating enhanced blood flow and the development of an erection [4,12,19]. PDE5i enhance this process by maintaining higher cGMP levels, thereby improving erectile function [4,12,19]. The clinical efficacy of PDE5i has been extensively validated across a wide range of demographic groups and clinical contexts. Sildenafil, tadalafil, and vardenafil are among the most extensively researched and commonly prescribed PDE5i. The efficacy of these drugs has been demonstrated in numerous randomised controlled trials and they are, therefore, the first-line treatment for the majority of men with ED. These drugs are efficacious in patients with ED of varying aetiologies, including those with vascular, neurological, and psychological origins, as well as in difficult-to-treat populations, such as diabetic men [12,19]. It should be noted that, despite their effectiveness, PDE5i are not suitable for all individuals. These medications are contraindicated in patients who are using nitrates for the treatment of coronary artery disease, due to the potential risk of severe hypotension [4,12]. Furthermore, it is recommended that caution be exercised in patients with cardiovascular disorders, as sexual activity itself may pose a risk. Emerging research continues to explore the potential for new PDE5i with improved efficacy and safety profiles. PDE5i remain a fundamental component of the therapeutic arsenal for ED. Their role continues to expand with advances in our understanding of their mechanisms, effectiveness across different patient scenarios, and potential applications beyond traditional indications. Recent advancements in PDE5i have introduced newer agents that offer improved selectivity, faster onset of action, and better tolerability compared to traditional options like sildenafil, tadalafil, and vardenafil. Among these, avanafil and udenafil have shown promising results in clinical trials. Avanafil, a second-generation PDE5i, is characterised by its rapid onset of action (15 min) and higher selectivity for PDE5, resulting in reduced off-target side effects [21]. A significant improvement in erectile function was observed with avanafil (IIEF-EF: MD = 4.39, 95%, *p* < 0.001), as well as an increase in successful intercourse rates [21]. The SEP-3 study reported a relative risk (RR) of 2.30 (95% CI [2.01, 2.62], *p* < 0.001) for successful intercourse, while the study by Jiang et al. found that over 40% of patients using avanafil experienced successful intercourse, in contrast to the 22% observed in the placebo group [22]. Furthermore, a study by Tsai et al. reported that 87.5% of patients reported satisfaction with the avanafil treatment [23]. Udenafil, a long-acting PDE5i, allows for once-daily dosing, which has been shown to enhance treatment adherence [22]. A 2022 randomized controlled trial revealed that patients who received 75 mg of udenafil daily for 32 weeks exhibited significant improvements in their IIEF scores (22 or higher in 36.51% of patients) compared to those who received a placebo (13.04%, *p* = 0.021) [24]. Moreover, 82.54% of patients administered with udenafil reported a 25% or greater enhancement in erectile function [24]. In a separate study, Park et al. found that patients suffering from post-surgical ED experienced a significant improvement in their IIEF scores (4.8 ± 4.0 vs. 2.0 ± 1.7 in placebo, *p* < 0.05) after treatment with udenafil [25].

### 3.2. Regenerative Therapies

Regenerative therapies represent a pioneering approach to the treatment of ED, offering potential solutions for the restoration of erectile function through the regeneration and repair of affected tissue. These advanced therapies include stem cell treatments, platelet-rich plasma (PRP) injections, and gene therapy, which are designed to address the underlying causes of ED rather than merely managing the symptoms.

### 3.3. Stem Cell Therapy

Stem cell therapy entails the utilisation of stem cells for the purpose of regenerating damaged or diseased penile tissue [14,20,26]. This approach exploits the intrinsic capacity of stem cells to differentiate into diverse cell types and facilitate tissue repair [14,26]. It has been demonstrated that stem cell therapy can facilitate the restoration of normal erectile function by promoting the regeneration of endothelial and smooth muscle cells, thereby enhancing blood flow and vascular function within the penis [26,27]. Clinical trials have yielded encouraging results, with improvements in erectile function and minimal adverse effects [26,27]. However, a lack of sufficient data precludes the formulation of an optimal protocol and dosage. Consequently, further research is imperative [26,27].

### 3.4. PRP Therapy

PRP therapy employs the injection of a concentrated platelet solution to accelerate the healing of injured tendons, ligaments, muscles, and joints. This approach has been demonstrated to be effective in numerous studies [20,28,29]. In the context of ED, PRP injections are believed to stimulate the repair and regeneration of erectile tissue [20,29]. This is achieved through the release of growth factors that promote cell proliferation and tissue recovery. Studies have reported improvements in penile function, increased blood flow, and enhanced erectile response following PRP therapy, indicating that it may be a promising option for patients seeking non-pharmacological treatment alternatives [20,28,29].

### 3.5. Gene Therapy

Gene therapy represents a novel approach to the treatment of ED, offering the potential to target and modify the underlying genetic factors that contribute to this condition [30,31]. In contrast to conventional treatments, which typically address the symptoms of ED, gene therapy aims to rectify or enhance the biological mechanisms that are essential for erectile function [30,31]. Gene therapy for ED entails the direct injection of genetic material into penile tissue, with a particular focus on corporal smooth muscle cells [30,31]. This approach has the advantage of minimising systemic exposure and potential side effects [30]. Given the relatively low turnover rate of corporal vascular smooth muscle cells, introduced genes have the potential to express therapeutic proteins for extended periods, thereby enhancing long-term efficacy [30,31]. Gene therapy can be designed to target specific molecular pathways involved in erectile function, thereby offering the possibility of customising treatments based on individual pathophysiology [30]. Despite the promising theoretical framework, the practical application of gene therapy in the treatment of ED has progressed at a slow pace [30]. The initial clinical trials utilising gene therapy for ED entailed the transfer of the hSlo gene, which encodes for the Maxi-K potassium channel, a pivotal modulator in the smooth muscle cell function of the penis [30]. The preliminary findings from clinical trials indicate that gene therapy may be a promising avenue for enhancing erectile function, without significant adverse effects [30,31]. Nan et al. demonstrate that the administration of glial growth factor 2 (GGF-2) in patients with cavernous nerve injury can restore the integrity of the cavernous nerve and reinstate erectile function [32]. Nevertheless, gene therapy faces a number of challenges. As ED is not a life-threatening condition, the safety standards for gene therapies in this context are exceptionally high. The therapy must demonstrate a robust safety profile in order to advance further in clinical trials. Approval for new gene therapies, particularly for non-life-threatening conditions such as ED, necessitates the presentation of extensive data to demonstrate that the benefits outweigh the risks [30,31].

### 3.6. Challenges and Ethical Considerations

Despite the potential benefits, regenerative therapies present a number of challenges. The intricacy of these treatments necessitates the utilisation of sophisticated delivery techniques and meticulous monitoring to guarantee the safety and efficacy of the procedure. Furthermore, the considerable financial investment required for the development and administration of regenerative therapies can restrict accessibility for a significant proportion of patients. Ethical considerations also assume paramount importance, particularly with regard to the assurance provided by informed consent and the management of patient expectations. As these therapies are still in the early stages of development, the availability of long-term safety and effectiveness data is limited, necessitating the continuation of research and clinical trials to establish robust evidence in support of their use [10].

### 3.7. Shock Wave Therapy

Shock wave therapy has emerged as a promising non-invasive treatment for ED, particularly for patients who are unresponsive to conventional pharmacological treatments. This therapy employs low-intensity shock waves to stimulate angiogenesis (the formation of new blood vessels) and enhance blood flow in penile tissues, which are essential for achieving and maintaining an erection [20,29,33,34]. The mechanism of action of shock wave therapy involves the delivery of focused low-intensity shock waves to the penile tissue [34,35]. The shock waves induce microtrauma in vascular endothelial cells, which in turn stimulates the release of angiogenic factors, such as the vascular endothelial growth factor (VEGF) and fibroblast growth factor [29,34,35]. These factors promote the formation of new blood vessels and rejuvenate the blood flow to the penis, enhancing erectile capacity [29,35]. Clinical studies have demonstrated that shock wave therapy can markedly enhance erectile function in males with vasculogenic ED, a condition characterised by ED caused by blood vessel dysfunction [34,35]. One of the primary advantages of shock wave therapy is its capacity to potentially restore natural erectile function and reduce the reliance on ED medications. Furthermore, the non-invasive nature of the treatment renders it an appealing prospect for a considerable number of patients. Nevertheless, the long-term efficacy and safety of shock wave therapy remain subjects for further investigation. While preliminary outcomes are encouraging, additional research is necessary to establish standardised treatment protocols and ascertain the optimal intensity and frequency of shock waves required to achieve optimal outcomes [29,34].

### 3.8. Physiotherapy

The use of physiotherapy as a treatment for ED is becoming increasingly prevalent due to its advantages as a non-invasive, non-pharmacological approach. The utilisation of pelvic floor muscle exercises (PFMEs), manual therapies, and patient education plays a pivotal role in the enhancement of sexual function and quality of life for men. The pelvic floor is a complex structure comprising muscles, ligaments, and connective tissues that are responsible for the coordination of urine and faeces release [36]. Moreover, the contraction and relaxation of the aforementioned muscle plays a role in erectile function and ejaculation [36,37]. It has been demonstrated in research studies that the contraction of the pelvic floor muscles results in increased penile rigidity in patients experiencing ED [37,38]. The bulbospongiosus and ischiocavernosus muscles are of particular importance in the context of erectile function [38,39]. These muscles enhance penile rigidity by compressing the deep dorsal vein in the penis, thereby restricting venous outflow and maintaining an erection [39]. Manual therapies that encompass techniques designed to normalise muscle tone and improve muscle relaxation may also prove beneficial in the management of ED, particularly when combined with pelvic floor muscle exercise [25]. Pan et al. posit that pelvic floor muscle exercises utilising resistance bands may prove beneficial for patients with ED following radical prostatectomy [40]. Moreover, a substantial body of research has demonstrated that regular pelvic floor muscle exercise can enhance erection quality by strengthening the muscles responsible for stabilising and maintaining an erection [36,37,41,42]. Another method that can be combined with PFME is biofeedback [43]. Biofeedback is used to enhance patients’ awareness of how pelvic floor muscles function and to optimise the control and strength of these muscles [38,43,44]. Chen et al. demonstrate that biofeedback, in conjunction with low-intensity pulsed ultrasound, can reduce clinical symptoms in patients with ED [45].

### 3.9. Impact of Dietary Patterns

The relationship between diet and ED is primarily determined by the impact of dietary choices on the patient’s pro-inflammatory state, vascular health, hormonal balance, and overall physiological well-being. These factors are essential for maintaining erectile function [46,47]. The relationship between cardiovascular health and erectile function is well-documented in the literature [48,49,50]. ED frequently serves as an early indicator of potential cardiovascular issues, as both conditions are associated with shared risk factors, including hypertension, hyperlipidaemia, and diabetes [47,48,49,50,51]. A diet high in saturated fats and low in essential nutrients can result in endothelial damage, leading to the development of atherosclerosis. This is a narrowing and hardening of the arteries that restricts blood flow throughout the body, including to the penile tissues, which can impair erectile function [15,51]. In contrast, diets comprising fruits, vegetables, whole grains, and healthy fats have been demonstrated to enhance cardiovascular well-being and diminish oxidative stress and inflammation, thereby facilitating enhanced erectile capacity [15,51]. Furthermore, dietary habits may influence hormonal levels, which are known to play a pivotal role in sexual function [52,53]. A reduction in testosterone levels is associated with a decline in sexual desire and the development of ED. Nutrient-rich diets that include foods high in zinc and vitamin D have been demonstrated to maintain and even boost testosterone levels, thereby supporting erectile function and overall sexual health. Antioxidants play a pivotal role in maintaining endothelial health, which is indispensable for the effective synthesis of NO, a molecule that is crucial for erectile function [15,51,54]. NO is a vasodilator that relaxes smooth muscle in the penis, facilitating the blood flow necessary for achieving and maintaining an erection [51]. Diets high in antioxidants, which are abundant in fruits, vegetables, nuts, and seeds, help combat oxidative stress and protect vascular health, which can enhance NO availability and support erectile function [15,47,51].

#### The Mediterranean Diet

**Figure 2:**
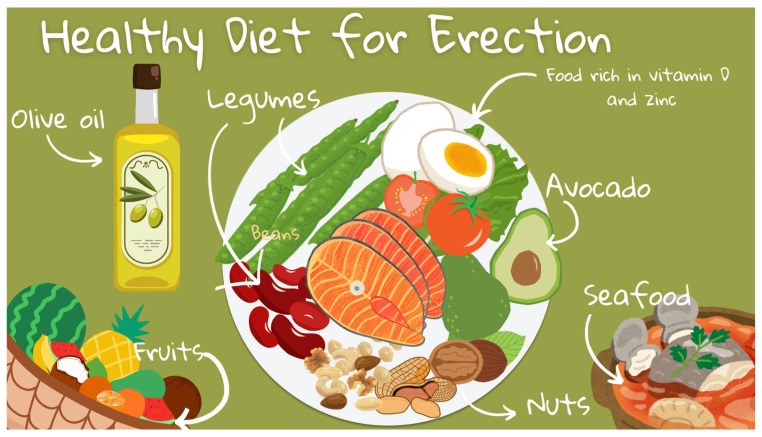
Diets that influence erectile function.

The Mediterranean diet, which is renowned for its beneficial effects on cardiovascular health, has also been identified as a dietary pattern that can improve ED [47,51,53,55]. The diet places an emphasis on the consumption of fruits, vegetables, whole grains, legumes, nuts, and olive oil, with moderate amounts of fish and poultry, and a limited intake of red meat and processed foods [15,51]. The Mediterranean diet has been extensively documented for its role in promoting cardiovascular health, primarily through the reduction of cardiovascular risk factors, such as high blood pressure, cholesterol, and inflammatory markers [47,51,53]. Given that vascular health constitutes a pivotal element of erectile function, the diet’s capacity to enhance blood flow and endothelial function directly impacts the ability to sustain an erection [47,51]. A number of studies have demonstrated a correlation between adherence to the Mediterranean diet and a lower prevalence of ED [47,51,55]. The Mediterranean diet represents a promising and practical approach to managing ED through dietary modification. Further research and clinical trials will help to establish the Mediterranean diet as a foundational treatment strategy for ED, emphasising the importance of holistic, lifestyle-oriented approaches in managing health conditions. A summary of the diets that influence erectile function is presented in Figure 2.

### 3.10. Herbal Supplements

The use of herbal supplements as a means of treating ED and enhancing sexual health is a practice that has been observed across various cultures for a considerable period of time. As interest in holistic and non-pharmacological approaches grows, the use of botanicals in ED treatment is becoming increasingly popular. This is due to their perceived safety profile and potential health benefits. However, the effectiveness and mechanisms of action of these supplements vary, necessitating careful consideration and scientific validation. Several herbs are commonly associated with the treatment of ED, each possessing unique properties that may contribute to their efficacy.

### 3.11. Ginseng

It has been demonstrated that ginseng can enhance erectile function by facilitating penile blood flow through the enhancement of NO synthesis and release [56,57]. Furthermore, a limited number of studies have indicated a favourable impact of ginseng on testosterone levels [57,58]. The available evidence suggests that ginseng may have a beneficial effect on erectile function in patients with ED (ED) [57,59,60]. However, research by Lee et al. indicates that ginseng has only a minimal impact on the improvement of erectile function [56]. While ginseng’s properties appear promising, further research is required to confirm these findings.

### 3.12. Yohimbine

Yohimbine is an indole alkaloid that has been employed as an aphrodisiac, with the objective of improving erectile function [61]. Yohimbine exerts its effects by blocking alpha-2 adrenergic receptors, which can facilitate increased blood flow to the penis and promote erection [61,62] Furthermore, yohimbine has been demonstrated to possess antidiuretic, anti-adrenergic, mydriatic, and serotonin antagonist effects [61]. In Germany and Canada, yohimbine has been employed in the treatment of ED for over twenty years. However, yohimbe has been associated with a range of adverse effects, including increased heart rate and blood pressure, chest pain, and palpitations, underscoring the importance of its use under medical supervision [61].

### 3.13. Horny Goat Weed (Epimedium)

Horny goat weed, also known as epimedium or Yinyanghuo, contains icariin and icariside II, which are flavonoids with diverse biological properties. These include anti-inflammatory, antioxidant, anti-osteoporotic, anticancer, and anti-aging effects, as evidenced by references [63,64,65]. Beyond these general health benefits, flavonoids, particularly icariin and icariside II, play a crucial role in vascular health and endothelial function, which are fundamental to erectile function. Flavonoids have been the subject of extensive research due to their potential to counteract oxidative stress and inflammation, both of which are well-established contributors to endothelial dysfunction and ED (ED) [66]. Icariside II has been demonstrated to enhance endothelial function by upregulating eNOS expression and stimulating the proliferation of the cavernous endothelium. It is, therefore, possible that horny goat weed may assist in maintaining adequate blood flow and erection quality [63,64]. In addition, icariside II has been shown to mitigate endothelial cell damage caused by high glucose levels, which is particularly important for individuals with diabetes, a condition strongly associated with ED [64]. By preserving endothelial health, these flavonoids not only support normal erectile function, but may also serve as a protective agent against diabetic ED [66]. A large-scale study involving 25,096 men found that higher flavonoid intake was associated with a significant reduction in the incidence of ED, with specific subclasses, such as flavones and flavanones, reducing ED risk by 11–16% [67]. Moreover, a study by Mykoniatis et al. revealed that men who consumed at least 50 mg of flavonoids per day exhibited a 32% lower risk of developing ED [68]. Furthermore, Yinyanghuo has been shown to contribute to NO release from the corpus cavernosum, leading to relaxation of the smooth muscle and, consequently, an erection [63]. This NO-mediated pathway is the same mechanism targeted by PDE5i, such as sildenafil, suggesting that the flavonoids in horny goat weed could serve as a natural alternative or adjunct to these conventional treatments [66]. The ability of flavonoids to promote endothelial health, enhance NO production, and counteract oxidative damage, further solidifies their importance in ED prevention and management. However, despite these promising findings, the exact mechanisms of epimedium remain unclear, necessitating further research [64].

### 3.14. Surgical Treatment

Surgical intervention, particularly penile prosthesis implantation, represents a well-established treatment for ED that has proven unresponsive to conservative therapies. This approach is frequently employed in cases of vasculogenic ED and ED resulting from radical prostatectomy [69]. The surgical treatment provides a reliable and effective solution for restoring erectile function, with high satisfaction rates among patients and their partners [70]. There are numerous varieties of penile prostheses [70]. One such option is that of inflatable penile prostheses (IPPs), which encompass two-piece and three-piece systems. Three-piece devices are the most commonly utilised due to their capacity to emulate the nuances of both erection and flaccidity with remarkable fidelity. Furthermore, inflatable prostheses offer superior cosmetic outcomes in comparison to alternative implant alternatives [70]. An alternative option is malleable prostheses, which are simpler devices that maintain a rigid state. However, they are less common due to lower patient satisfaction compared to inflatable options [70,71]. In the majority of studies, patient satisfaction rates exceed 70%, with some studies reporting satisfaction levels as high as 90% for inflatable penile prostheses [72,73]. Satisfaction rates are influenced by improvements in sexual function, emotional well-being, and quality of life following surgery [72,74]. Psychological benefits, including improved self-esteem and confidence, were consistently identified as advantages of the procedure [73]. Partner satisfaction rates vary considerably, from 50% to 90%, reflecting differences in expectations, outcomes, and study methodologies [73]. Partners of patients with inflatable devices tend to report higher satisfaction than those with two-piece devices [74]. However, a decline in partner satisfaction over time has been observed, often linked to unmet expectations, difficulties with prosthesis use, or residual discomfort [75].

### 3.15. Vacuum Erection Devices

Vacuum erection devices (VEDs) have been demonstrated to be an efficacious intervention for the management of ED in patients who have undergone robot-assisted radical prostatectomy [76]. A total of 78% of users who continued treatment reported that the device was effective in achieving erections sufficient for penetration [76]. The compliance rate for this non-invasive therapy was 70.9%, with 70.9% of men who initiated therapy continuing to use the VEDs [76]. A high level of user satisfaction was observed, particularly among those who continued long-term usage [76]. Among the 29% of patients who discontinued VED use, the primary reasons included perceived ineffectiveness (37%) and discomfort in regard to the device (29%) [76]. VED therapy was well-tolerated, with no significant adverse events reported in the studies referenced [76,77]. The utilisation of an additional VED prior to penile prosthesis surgery has been demonstrated to enhance surgical outcomes and patient satisfaction [68]. The preoperative administration of a VED (10–15 min daily for 30 days) has been shown to significantly augment the flaccid stretched penile length by an average of 0.8 cm [77]. Patients have also reported more expedient corporal dilatation during surgery, which facilitated the implantation process [77].

**Figure 3:**
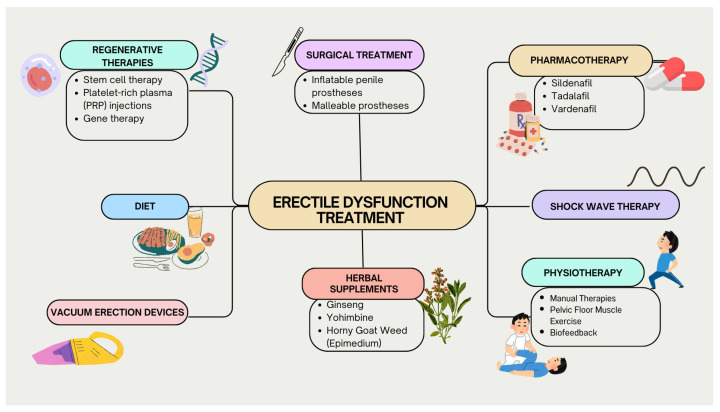
Treatment methods for ED.

Various treatment methods for ED are shown in Figure 3.

### 3.16. Psychological Treatment

Psychological treatments for ED offer a range of effective interventions that address the emotional and cognitive factors underlying the condition. Cognitive behavioural therapy (CBT) has been the subject of extensive research due to its ability to reduce performance anxiety and restructure negative beliefs about sexual performance. A randomised controlled trial conducted during the pandemic revealed that men receiving CBT in conjunction with PDE5i exhibited sustained enhancements in erectile function, while those who used PDE5i as monotherapy experienced no further progress [78]. The International Index of Erectile Function (IIEF) scores remained significantly higher in the CBT group compared to the monotherapy group [78]. A randomised controlled trial conducted during the pandemic of severe acute respiratory syndrome (SARS-CoV-2) found that online CBT significantly improved erectile functioning, self-esteem, and reduced depression and anxiety among men with nonorganic ED [79]. Erectile function scores improved significantly after CBT. Before treatment, the IIEF score was 12.3, and after treatment, it was 19.6 (*p* < 0.05) [80]. A pilot study revealed that cognitive behavioural sex therapy (CBST) was as efficacious as sildenafil (Viagra) in the treatment of nonorganic ED (NOED) in young men, with CBST resulting in substantial enhancements in erectile function, and reductions in the severity of the dysfunction and depressive symptoms [79]. Sexual therapy and couples therapy have also been shown to play a pivotal role by enhancing communication and intimacy between partners. The European Society of Sexual Medicine emphasises that integrating partners into therapy can enhance treatment adherence and satisfaction [81]. Additionally, mindfulness-based interventions (MBIs) have garnered attention as a method to alleviate anxiety and cultivate present-moment awareness. Research findings indicate that MBI can substantially enhance self-esteem (from 22.67 to 30.43 on the Rosenberg scale) and reduce anxiety levels (from 11.13 to 8.36) in men with ED [80]. Overall, the integration of psychological approaches, including CBT, sexual and couples therapy or mindfulness, has been shown to enhance ED treatment outcomes and provide long-term benefits beyond medical interventions alone.

## 4. Conclusions

This review examined the efficacy of disparate independent treatments for ED, underscoring the necessity for a personalised, multifaceted approach. A diet that is conducive to cardiovascular health, such as the Mediterranean diet, which places an emphasis on the consumption of fruits, vegetables, whole grains, and healthy fats, has been demonstrated to significantly enhance endothelial function and reduce the prevalence of ED. PDE5i, including sildenafil, tadalafil, vardenafil, avanafil, and udenafil remain the primary treatment option for ED. Of particular note is the efficacy of udenafil in the long term, particularly in patients suffering from post-prostatectomy and post-surgical ED. These medications effectively enhance erectile function, yet they are not suitable for all patients. It is imperative that personalised pharmacotherapy is considered, taking into account individual health profiles and contraindications. Nutritional and herbal supplements, including ginseng, yohimbine, and horny goat weed, have demonstrated potential in regard to improving erectile function. However, the evidence is inconclusive, and further high-quality trials are necessary to establish standardised dosages, safety, and efficacy of these supplements. Clinicians should guide patients on the cautious use of supplements, considering potential interactions and side effects. Innovative regenerative therapies, such as stem cell therapy, PRP injections, and gene therapy, appear to have promising results in terms of restoring erectile function. Nevertheless, high costs, complexity, and the need for further research limit their widespread application. It is imperative that clinical trials are conducted in order to facilitate the advancement of these therapies. Non-invasive shock wave therapy has been demonstrated to hold considerable promise for the treatment of vasculogenic ED (ED), through the promotion of angiogenesis and the enhancement of penile blood flow. Although the initial studies are encouraging, further research is required to standardise treatment protocols and confirm the long-term benefits. A non-pharmacological approach to improving erectile function is offered by physiotherapy, which encompasses pelvic floor muscle exercises, manual therapies, and biofeedback. It is recommended that physiotherapy is incorporated into the management of ED, particularly for patients who prefer non-invasive treatments. Nevertheless, further research is required in order to develop a specific rehabilitation protocol. Psychological interventions, including CBT, mindfulness-based interventions (MBIs), and couples therapy, have been shown to play a critical role in enhancing treatment adherence, reducing performance anxiety, and improving patient outcomes. Future studies should explore how integrating psychological therapy with medical treatments influences long-term patient satisfaction. Surgical interventions, particularly penile prosthesis implantation, are an effective treatment option for patients who have not responded to other treatments. The use of inflatable penile prostheses has been found to result in high levels of satisfaction among both patients and their partners, with satisfaction rates often exceeding 70–90%. Surgical methods provide a reliable solution for restoring erectile function, with the additional psychological benefits of improved self-esteem and quality of life. VEDs represent a non-invasive alternative, particularly beneficial for post-prostatectomy ED patients. VEDs are a valuable tool in ED management. The preoperative use of VEDs, before penile prosthesis surgery, has been demonstrated to enhance surgical outcomes, including improved penile length and ease of implantation. In conclusion, the management of ED requires a comprehensive, personalised approach. It is reasonable to posit that optimal outcomes will be achieved through the implementation of personalised treatment plans that address both the symptoms and the underlying causes of ED. The combination of different treatment methods allows healthcare providers to offer effective, holistic care, which improves erectile function and enhances the quality of life for men with ED. To achieve this, it is necessary to investigate the potential synergistic effects of combining different therapies. These could include the use of PDE5 inhibitors in conjunction with physiotherapy, shock wave therapy in combination with regenerative medicine, and other similar approaches. In order to evaluate this kind of research, the Sexual Encounter Profile (SEP) and the Patient Global Impression of Improvement (PGI-I) could be useful tools. Another valuable avenue for research would be to assess the emotional and relational impacts of different treatment methods, which would allow us to explore the role of psychological counselling and partner involvement in treatment outcomes. In order to predict the kind of research that would be beneficial in this regard, the Sexual Quality of Life Questionnaire (SQoL-M/F) and the Dyadic Adjustment Scale (DAS) could be employed.

## Data Availability

The data presented in this study are available on request from the corresponding author. The data are not publicly available due to privacy restrictions.

## Supplementary Materials

The following supporting information can be downloaded at: https://www.mdpi.com/article/10.3390/jcm14072386/s1, Table S1: Summary of the reviewed literature.
